# Neuroprotective Effects and Mechanisms of *Alpiniae oxyphyllae* Fructus, a Medicinal and Edible Homologous Herb: Research Advances

**DOI:** 10.3390/ijms26136230

**Published:** 2025-06-27

**Authors:** Yongyi Wei, Ke Gao, Yidong Sun, Qing Sheng, Jianjun Guo

**Affiliations:** College of Life Sciences and Medicine, Zhejiang Sci-Tech University, Hangzhou 310018, China; 2022332864086@mails.zstu.edu.cn (Y.W.); 2022332864026@mails.zstu.edu.cn (K.G.); 2023332864030@mails.zstu.edu.cn (Y.S.)

**Keywords:** neurological diseases, *Alpinia oxyphylla* Miq., neuroprotection, mechanisms of action

## Abstract

Neurological diseases, which include various neurodegenerative disorders, not only impair patients’ physical health but also impact their psychological and social functions. It is particularly urgent to seek effective prevention and treatment strategies for neurological diseases. *Alpiniae oxyphyllae* Fructus (AOF), a traditional Chinese medicinal herb, has been widely used in treating urinary, digestive, and neurological disorders. Contemporary medical research has demonstrated that AOF exerts neuroprotective effects through multiple mechanisms, including by inhibiting neuronal apoptosis, alleviating neuroinflammation, reducing oxidative stress, and regulating nerve cell dynamic balance. In recent years, substantial advancements have been achieved in investigations concerning the neuroprotective effects and underlying mechanisms of AOF, alongside significant breakthroughs in its clinical applications. This review systematically summarizes the neuroprotective effects of AOF and delineates its clinical applications, thereby offering valuable reference and guidance for the prevention and treatment of neurological diseases using AOF.

## 1. Introduction

*Alpiniae oxyphyllae* Fructus (AOF, Yizhiren in Chinese) is the dried mature fruit of *Alpinia oxyphylla* Miq., a plant species belonging to the Zingiberaceae (ginger) family. As a medicinal food homologous substance, its consumption can be traced back to ancient times [1]. In 2012, it was officially listed in China’s “Catalogue of the Substances Traditionally Considered as both Food and Chinese Medicine.” Classical Chinese pharmacopeias have long documented its therapeutic properties. Traditionally, AOF is characterized as having a “warm” nature and “pungent” flavor, and is commonly used to warm the spleen (such as relieving diarrhea due to spleen coldness) and tonify the kidney (like a stabilizing essence to address sperm leakage or frequent urination) [2,3,4,5]. Its culinary applications are diverse, including preparation as preserved fruits, candied snacks, medicinal wines, congee, herbal soups, and teas [2]. Modern pharmacological investigations have demonstrated that AOF is abundant in vitamins B1, B2, and E, along with diverse amino acids and calcium, making it a high-protein, low-cholesterol dietary ingredient. Furthermore, it contains multiple bioactive constituents, including flavonoids, diarylheptanoids, sesquiterpenes, and volatile oils. These constituents exhibit a wide range of pharmacological activities, including antibacterial, antiviral, renoprotective, antitumor, cardioprotective, gastro regulatory, antioxidant, anti-aging, memory-enhancing, and neuroprotective effects.

Neurological diseases represent a category of intricate disorders affecting both the central and peripheral nervous systems (PNS), encompassing neurodegenerative diseases (e.g., Alzheimer’s disease, AD; Parkinson’s disease, PD), and neurodevelopmental disorders (e.g., autism spectrum disorder), as well as neuroinflammation and nerve injury [6,7,8,9]. These diseases not only severely impair patients’ cognitive, motor, and sensory functions but also place substantial burdens on society and families. With the intensification of population aging, the incidence of neurological diseases is showing an upward trend [10]. Therefore, there is an urgent need to develop safe and effective broad-spectrum therapeutic agents to alleviate or treat neurological diseases. In light of the relevant research on AOF, scientists are actively exploring its mechanisms and therapeutic potential in treating neurological diseases. This article aims to systematically review the neuroprotective effects and underlying mechanisms of AOF, a substance that is both a medicinal agent and a functional food.

## 2. Materials and Methods

Bibliometrics is an interdisciplinary science that uses mathematical and statistical methods to quantitatively analyze various knowledge carriers. By conducting qualitative and quantitative visual analyses of a large number of relevant documents in a specific field, bibliometrics aims to explore the relationships, structure, intersections, and evolutionary trends among knowledge units within that scientific domain [11,12].

VOSViewer is a tool that generates knowledge maps by analyzing literature citations and keyword co-occurrences. It uses colors, node sizes, and density color patches to differentiate research clusters, indicate the intensity of research activity, and highlight core areas, respectively. This enables researchers to intuitively understand hotspots, developmental paths, and collaborative networks within a field [13].

In recent years, international research on AOF has advanced significantly, particularly in neuroprotective diseases, with a growing number of related papers. Consequently, this study will employ VOSViewer (1.6.20.0) to analyze AOF in the field of neurodegenerative diseases.

This study retrieved data from the PubMed and Web of Science databases without time restrictions. After multiple trials, the search strategy “ALL = (Alpinia oxyphylla)” was chosen to ensure comprehensiveness, accuracy, and authority. Specifically, the Web of Science Core Collection was selected for Web of Science indexing.

**Figure 1:**
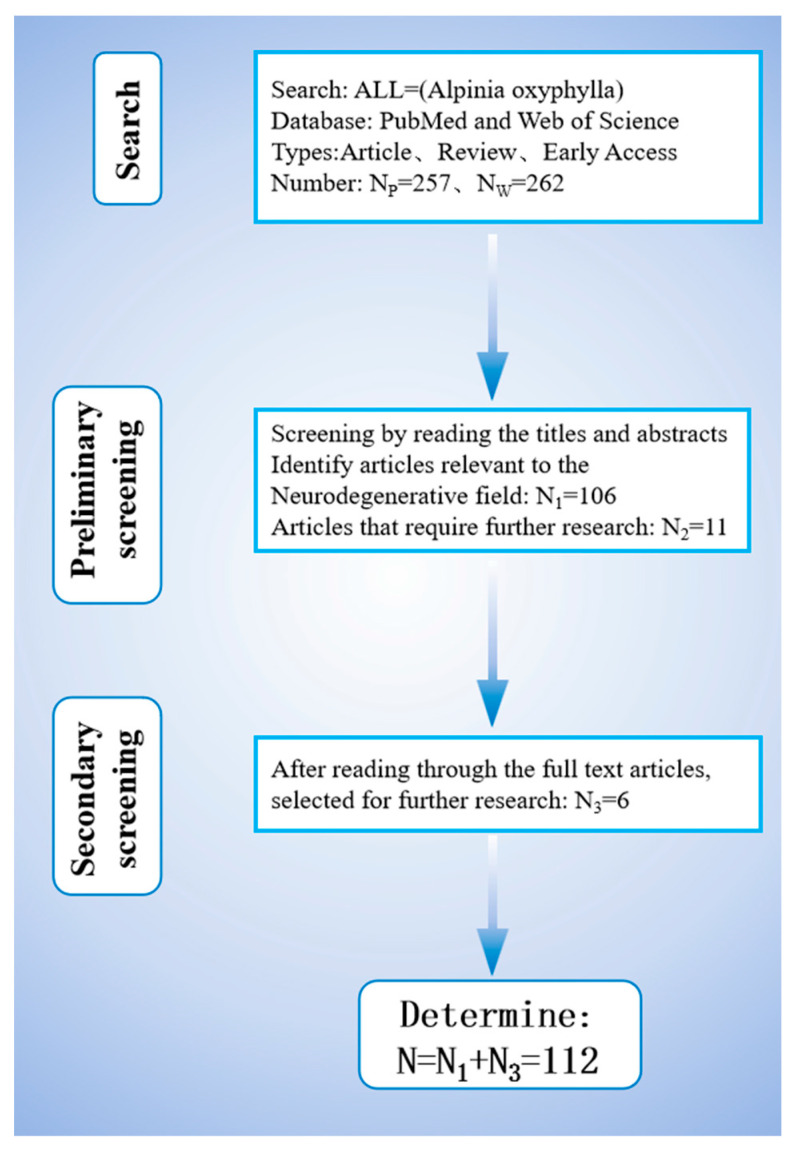
Flowchart of literature screening.

The search identified 257 and 273 studies on PubMed and Web of Science, respectively. Articles categorized as article, review, or early access were chosen, and those related to neurological diseases were shortlisted via title and abstract screening. After merging and deduplicating the datasets, 106 studies remained, with 11 requiring further screening. By reading the full texts of these 11 studies, a total of 6 studies related to neurological diseases were screened out. Figure 1 illustrates the screening process.

**Figure 2:**
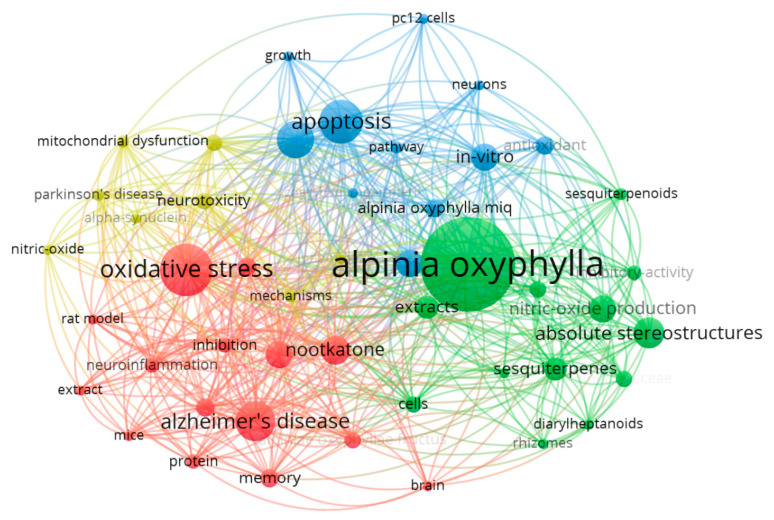
Relationship diagram of literature keywords created by VOSViewer software.

To gain a deeper understanding of how different knowledge units interconnect, we conducted a keyword co-occurrence analysis using VOSViewer on the 112 selected studies (Figure 2). The analysis settings were as follows: the type of analysis, unit of analysis, and counting method are “Co-occurrence”, “All keywords”, and “Full counting”, respectively. From 659 keywords, 47 with the highest occurrence rates (each appearing at least 5 times) were selected. In Figure 2, the keyword relevance network diagram is divided into four groups (different colors for different nodes). Lines indicate collaboration, with their thickness reflecting the strength between two nodes. Nodes represent keywords, with node size being proportional to keyword frequency in the literature [14].

## 3. Chemical Composition and Pharmacological Basis of AOF

**Figure 3:**
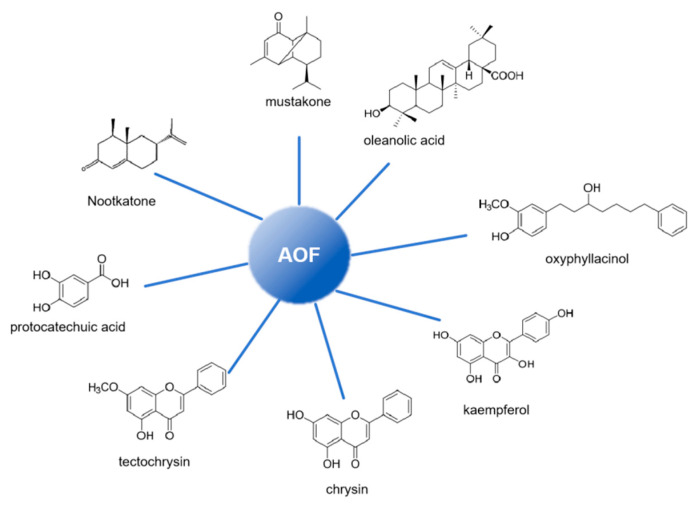
Main components in AOF.

Building upon the well-documented anti-inflammatory and antioxidant properties of AOF, scientists have integrated the pathogenic mechanisms of neurological diseases with research insights from Traditional Chinese Medicine (TCM) regarding AOF, aiming to address intractable neurological diseases associated with neuroinflammation and neuronal oxidative stress. Through systematic experimental investigations, the primary bioactive components of AOF have now been successfully identified [15,16] (as shown in Figure 3).

Recent studies have highlighted the potential of AOF as a valuable resource for natural drug development, driven by its abundant bioactive phytochemicals and broad pharmacological activities. Specifically, the volatile oil constituents exhibit inhibitory effects on pathogens like *Staphylococcus aureus* and *Escherichia coli*. The polysaccharide fractions contribute to enhance immune function, while the flavonoid components exert antioxidant effects by scavenging free radicals [5,17,18].

## 4. Neuroprotective Effects and Mechanisms of AOF

In recent years, the neuroprotective mechanism of AOF has been extensively investigated [19,20,21]. Modern pharmacological studies have progressively elucidated the intervention capabilities of its active components, such as nootkatone (NKT), chrysin, and protocatechuic acid (PCA), in critical pathological processes, including neuroinflammation, oxidative stress, and the promotion of cell proliferation (Table 1). These insights have been achieved through advanced technologies like molecular docking, transcriptomics, and metabolomics [5,22].

### 4.1. The Neuroprotective Effect of AOF

#### 4.1.1. Anti-Inflammatory Effect

Inflammation serves as the body’s response to tissue damage, which can be elicited by physical trauma, ischemic injury, infection, exposure to toxins, or other contributing factors [32]. Neuroinflammation, as a special form of inflammation, can be jointly triggered by central pathogenic factors and peripheral inflammation. Microglia, as the main immune cells of the central nervous system (CNS), can be classified into M1 (pro-inflammatory and cytotoxic) and M2 (anti-inflammatory and neuroprotective) phenotypes based on their activation status. Persistent inflammatory responses can activate microglia to transform into M1 and intensify inflammation, thereby inducing neurodegenerative diseases. The initial stage that leads to this process is mainly driven by endogenous factors (such as protein aggregates), and peripheral inflammation can assist in the occurrence of neuroinflammation by disrupting the blood–brain barrier and promoting the increase in cytokines and immune cells in cerebrospinal fluid [33,34,35,36]. Consequently, identifying novel therapeutic targets and potential pharmacological agents for microglia-mediated neuroinflammation holds significant promise [37,38].

Dong et al. [23] conducted an in-depth investigation into the sesquiterpenoids in the fruits of AOF and their anti-inflammatory activities. Through bioactivity-guided isolation and purification, 38 sesquiterpenoids were successfully isolated from the crude extract of AOF. The study demonstrated that several of these compounds exhibited potent inhibitory effects on the secretion of cytokines (TNF-α and IL-6) and NO in lipopolysaccharide (LPS)-stimulated BV-2 microglial cells, thus effectively suppressing inflammatory responses. Xu et al. [24] found that AOF reduced NO and TNF-α levels while increasing IL-10 levels in BV2 cells, demonstrating its anti-inflammatory effects. Further studies revealed that chrysin in AOF binds to triggering receptor expressed on myeloid cells 2 (TREM2), regulating the PI3K/AKT/GSK3β pathway to exert neuroprotective anti-inflammatory effects. These findings support the traditional medicinal use of AOF and provide a basis for exploring its active components and mechanisms.

#### 4.1.2. Antioxidant and Antiapoptotic Effect

Oxidative stress refers to a state of imbalance between ROS and reactive nitrogen species (RNS) and the body’s antioxidant defenses, leading to ROS or RNS accumulation [39,40]. Under normal conditions, ROS act as signaling molecules regulating processes like cell proliferation, differentiation, and apoptosis. During oxidative stress, however, excessive ROS cause cellular damage through lipid peroxidation, protein oxidation, and DNA damage, impairing cell structure and function [41,42,43,44,45].

Oxidative stress plays a crucial role in neurodegenerative diseases. Neurons, with high metabolic demands and low antioxidant capacity, are vulnerable to oxidative damage [46,47]. This stress causes mitochondrial dysfunction, reducing adenosine triphosphate (ATP) production and disrupting calcium balance, leading to apoptosis [48,49]. In AD, it not only enhances the β-site amyloid precursor protein cleaving enzyme 1 (BACE1)-mediated cleavage of amyloid precursor protein (APP) to generate aggregation-prone Aβ_1–42_ and promote Aβ aggregation, but also activates stress kinases, particularly the p-JNK pathway, to drive tau hyperphosphorylation. Meanwhile, Aβ aggregates induce mitochondrial dysfunction and the M1 polarization of microglia, thereby exacerbating ROS production, while phosphorylated tau further disrupts mitochondrial dynamics, forming a positive feedback loop in which oxidative stress, Aβ, and tau pathology mutually deteriorate. This interplay not only accelerates neuroinflammation via the nuclear factor kappa-B (NF-κB) signaling pathway, but also impairs neuronal energy metabolism, ultimately promoting neuronal loss [9,50]. In PD, oxidative stress damages dopaminergic neurons, impairing neurotransmitter function and causing movement disorders [51].

In response to the impact of oxidative stress on neurological diseases, Li et al. [25] investigated the therapeutic potential of AOF for AD and its mechanism. In vitro experiments showed that the AOF bioactive components PCA and NKT could significantly enhance the viability of PC12 cells treated with H_2_O_2_, reduce the apoptosis rate, and decrease the generation of ROS. It exerts antioxidant damage effects by enhancing the activities of SOD, CAT, and GSH-Px and maintaining mitochondrial membrane potential. In addition, it can also exert anti-apoptotic effects by activating the PI3K/AKT pathway. Meanwhile, Bian et al. [26] studied the novel compound oxyphylla A, extracted from AOF, and found that it could down-regulate the mRNA expressions of BACE1 and γ -secretase in N2a/APP cells and inhibit the production of Aβ. Moreover, it can inhibit the activity of key kinases that cause the excessive phosphorylation of tau protein by phosphorylating GSK-3β, and these conclusions have all been verified in AD model mice. Moreover, oxyphylla A can simultaneously improve cognitive function and motor disorders. Furthermore, Qi et al. [27] found that the combination of the active component NKT of AOF and schisandra glycoside could synergistically inhibit the release of inflammatory factors, such as IL-1β, IL-6, and TNF-α, induced by Aβ, as well as abnormal autophagy, further confirming the targeted intervention of its antioxidant stress and anti-apoptotic effects on the pathology of AD. The above studies indicate that AOF is not merely an antioxidant. Instead, through multi-component and multi-pathway mechanisms, it directly or indirectly regulates core pathological markers of AD such as Aβ and tau while eliminating ROS and inhibiting apoptosis, thereby exerting neuroprotective effects.

#### 4.1.3. Promoting Cell Migration and Proliferation

The mammalian brain is a network composed of various cells such as glial cells and neurons, which includes cellular connection structures like synapses, collectively forming a complex organ. Glial cells primarily consist of astrocytes, microglia, oligodendrocytes, and oligodendrocyte precursor cells, making up about half of all neural cells in the CNS of mammals. They mainly provide nutrition and support for neurons in the brain [52]. Neurons are a heterogeneous group of electrically active cells that form the framework of the brain’s intricate circuits. Neurons can be divided into those of the CNS and the PNS, with different anatomical structures and regenerative abilities between these two systems. In mammals, CNS neurons have limited regenerative capacity, whereas PNS neurons are myelinated and more easily regenerate [53,54].

Neurological diseases, such as AD, PD, or spinal cord injuries, involve neuron loss or dysfunction. Neuronal migration is a common process during embryonic development, but only a few types of neurons can migrate and differentiate in the CNS after adulthood. Those that do migrate follow trajectories formed by glial cells, primarily producing interneurons [55]. Specifically, cell migration guides new cells to injured sites, replenishing lost neurons and aiding neural network remodeling [56,57]. Migrated cells establish, restore, and secrete synaptic connections, signal transmission, and neurotrophic factors, respectively, supporting neuronal survival and differentiation [58]. For example, endogenous neural stem cells migrate from the subventricular zone to the damaged cerebral cortex, regulated by signaling molecules like chemokines, growth factors, and the extracellular matrix [59,60,61].

Cell proliferation, regulated by genes like cyclin D1 and cyclin-dependent kinase 4 (CDK4), increases nerve cell numbers, enhances neural plasticity, and supports neuron maturation, providing a critical foundation for nerve tissue repair [62,63]. During proliferation, biosynthesis and metabolism are enhanced to supply materials for neuronal growth and synapse formation. Increased cell volume and organelle numbers further boost nerve cell function [64,65]. Modulating pathways, such as RhoA/Rho-associated protein kinase [66,67] or mechanistic target of rapamycin (mTOR) [68], can improve axon regeneration. These processes work synergistically to promote nerve repair and functional recovery, offering a cellular basis for treating neurological disorders.

Schwann cells are support cells of the PNS that can differentiate into myelin sheaths of the PNS and proliferate and migrate to the ends of damaged nerves [69]. The migration of Schwann cells is crucial for axonal elongation and the remyelination of injured nerves [70,71]. A peripheral nerve injury typically activates Schwann cells and macrophages at the proximal end to synthesize neurotrophic factors, adhesion molecules, cytokines, and surface molecules that promote growth [72,73]. The ability of Schwann cells to promote peripheral nerve regeneration has increased interest in their use for PNS repair.

PCA, a bioactive component of AOF, was studied on RSC96 Schwann cell migration [28]. The 3-(4,5-dimethylthiazol-2-yl)-2,5-diphenyltetrazolium bromide (MTT) assay showed that PCA enhances cell proliferation. The Boyden chamber assay confirmed its promotion of both proliferation and migration. Western blot analysis revealed that PCA activates the ERK1/2, JNK1/2, and p38 pathways in the MAPK signaling cascade to cell migration.

Chang et al. [29] investigated the promoting effect of AOF extracts on nerve regeneration and its molecular mechanism using in vivo and in vitro experiments. In the in vivo experiment, varying concentrations of the AOF extract were injected into rat sciatic nerves. Analyses revealed increased levels of proliferating cell nuclear antigen (PCNA), cyclin A, and cyclin E, as well as enhanced insulin-like growth factor 1 (IGF-1) signaling pathway activity, as indicated by elevated protein levels of IGF-1, phosphorylated Akt (p-Akt), and B-cell lymphoma-extra large (BCl-xL). In the in vitro study, the AOF extract was applied to RSC96 Schwann cells, confirming consistent results. The findings suggest that AOF promotes nerve cell proliferation via the PI3K/Akt signaling pathway.

#### 4.1.4. Regulation of Metabolism

Improving nerve cell metabolism is a key strategy for treating neurological diseases. Enhanced metabolism can increase antioxidant capacity, eliminate excessive free radicals, reduce oxidative damage, inhibit lipid peroxidation and protein oxidation, and maintain cellular integrity [74,75,76]. Metabolic regulation can also activate autophagy, balancing protein synthesis and degradation to prevent abnormal protein aggregation, which is crucial for neurodegenerative diseases like AD and PD [77,78]. This multi-target approach provides a strong basis for slowing disease progression and repairing nerve function.

Using targeted and untargeted metabolomics approaches, Zhou et al. [30] found that AOF normalizes basal metabolites and modulates key metabolic pathways including sphingolipid, glycerophospholipid, fatty acid, and bile acid metabolism. AOF-treated AD model mice showed significant improvements in learning and memory, as confirmed by the Morris water maze test. These improvements correlated with the normalization of in vivo metabolites. Overall, AOF regulates behavior and reduces Aβ accumulation in the hippocampus and cerebral cortex, supporting its potential for clinical use in AD treatment.

Duncan et al. [31] investigated the effects of AOF using a rat model of AD with aggregated Aβ_1–42_ injected into the hippocampus. Through metabolomics analysis, it was revealed that AOF modulated nine brain metabolites and twenty-three plasma metabolites, primarily involving amino acid metabolism, lipid metabolism, and energy metabolism. Consequently, it was concluded that AOF restores most disordered and potentially disordered biomarkers to maintain the balance of associated pathways, thereby achieving the therapeutic goal of ameliorating AD.

### 4.2. The Neuroprotective Mechanism of AOF: Signal Pathway

**Figure 4:**
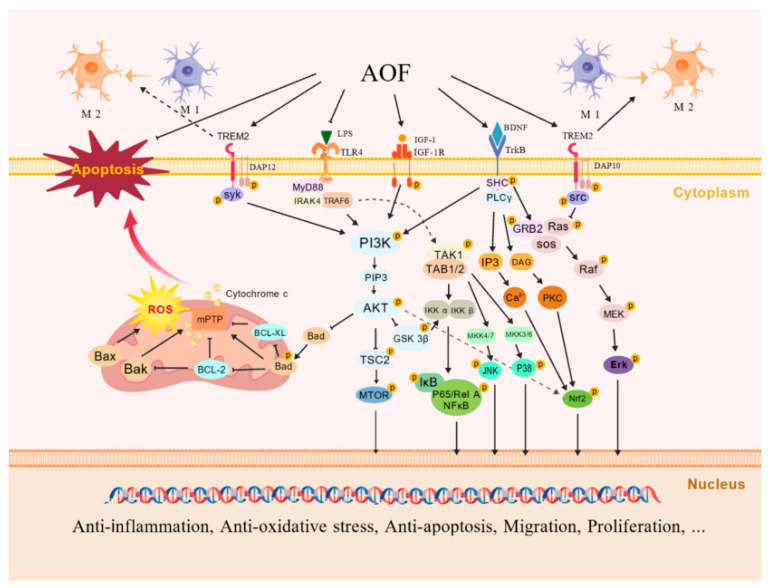
Neuroprotective mechanisms of AOF. Its components activate TREM2, recruit DAP12/DAP10, and inhibit the nuclear translocation of GSK3β and NF-κB by activating the PI3K/AKT pathway, and have anti-inflammatory responses by inhibiting the Ras/MEK/Erk pathway. Meanwhile, TREM2 mediates BDNF/TrkB to cause the following: inhibit the LPS-stimulated TLR4 signaling pathway; promote the M2 polarization of microglia; activate the IGF-1R/PI3K/AKT pathway; phosphorylate Bad; up-regulate Bcl-2 and Bcl-xL to resist apoptosis; regulate the cell cycle to promote cell proliferation; further activate Nrf2 to resist oxidation and inflammation; and activate ERK1/2/JNK/p38, promoting cell migration. Multi-pathway mechanisms synergistically exert anti-inflammatory, anti-oxidative, and anti-apoptotic effects, promoting cellular migration and proliferation. Created with BioGDP.com [80].

The results of the current research on the neuroprotective effect of AOF suggest that the improvement effect of AOF on neurological diseases may mainly be achieved through the regulation of the PI3K/AKT signaling pathway by its extracts and active components [24,29,79] (as depicted in Figure 4), specifically manifested as a multi-dimensional synergistic regulatory mechanism.

#### 4.2.1. TREM2/PI3K/AKT Signaling Pathway

TREM2 has been proven to be involved in the immune response and neuroinflammatory regulation of the CNS by regulating the activation, proliferation, and phagocytic functions of microglia in nerve cells, playing a key role in neurodegenerative diseases such as AD [81]. The active ingredient, chrysin, of AOF can activate TREM2. It recruits DNAX activation protein 12 (DAP12)/DNAX activation protein 10 (DAP10) adaptor proteins to trigger the activation of Syk and Src kinases, which, in turn, activates PI3K to generate PIP3 and recruits AKT to the cell membrane and phosphorylates it. Subsequently, AKT inactivates GSK3β by phosphorylating it, prevents the degradation of (i -κB), and inhibits the nuclear translocation of NF-κB, thereby reducing the transcription of pro-inflammatory genes such as TNF-α, although experiments have shown a decreased ratio of p-GSK3β/GSK3β [24,82,83,84]. The activated DAP10 can also interact with adaptor proteins, promote the recruitment of GRB2 and Sos1, and further inhibit the Ras/MEK/Erk pathway, thereby reducing the secretion of pro-inflammatory factors such as TNF-α and IL-1β [85]. Furthermore, the brain-derived neurotrophic factor (BDNF)/TrkB pathway can also be promoted by chrysin through TREM2-mediated signal transduction, and this effect can be eliminated by TREM2 siRNA. The TREM2/PI3K/AKT and TREM2/BDNF/TrkB pathways can jointly inhibit the pro-inflammatory signals mediated by Toll-like receptor 4 (TLR4) and the M1 phenotypic polarization of microglia induced by LPS, promote the M2 phenotypic polarization, reduce the secretion of NO and TNF-α, and increase the expression levels of anti-inflammatory factors such as IL-10 [24].

#### 4.2.2. IGF-1R/PI3K/AKT Signaling Pathway

In the nervous system, IGF-1 can bind to the IGF-1 receptor (IGF-1R) on the surface of nerve cell membranes, regulating the proliferation, differentiation, survival, synaptic plasticity, and energy metabolism of nerve cells and participating in neuroprotection, the formation of learning and memory, and the pathological regulation of neurodegenerative diseases [86]. The extracts and active components of AOF (such as PCA) can induce the phosphorylation of IGF-1R to activate PI3K. The accumulation of PIP3 leads to the activation of AKT. p-Akt up-regulates and down-regulates the expression of anti-apoptotic proteins Bcl-2 and Bcl-xL and the expression of pro-apoptotic protein Bax by phosphorylating Bad, respectively. It also inhibits the opening of mitochondrial permeability transition pores (mPTP) and the release of cytochrome c and reduces the activation of caspase-3, thereby inhibiting the apoptosis of nerve cells. On the other hand, AKT can stabilize cyclins D1, E, and A by phosphorylating GSK3β, promote the transition from the G1 phase to the S phase, and enhance the expression of PCNA, etc. [25,29]. Meanwhile, mTOR, as the most important substrate of AKT, can regulate cell growth and autophagy through p-AKT activation. Autophagy is closely related to oxidative stress, inflammatory response, and apoptosis. Continuously activated autophagy can lead to the death of neuronal cells and is involved in the occurrence of neurodegenerative diseases. The combined treatment of NKT and SCH not only regulates inflammation and apoptosis through PI3K/AKT but also inhibits the damage of autophagy to nerve cells by increasing the level of p-mTOR/mTOR [27].

#### 4.2.3. PI3K/AKT/Nrf2 Signaling Pathway

AOF extracts can activate Nrf2 by activating the PI3K/AKT signaling pathway and can up-regulate oxidases such as SOD, CAT, and GSH-Px to alleviate ROS damage in cells [25]. The active ingredient oxyphylla A was also verified in AD-simulated cell and mouse models to reduce the expression level of Aβ and decrease the phosphorylation of tau protein by activating AKT and inhibiting the activity of GSK3β. It also inhibits the expression of Kelch-like ECH-associated protein 1 (KEAP1) (which can enhance the ubiquitination of Nrf2 and promote its degradation), further activates Nrf2, prompts its translocation from the cytoplasm to the nucleus, and causes an antioxidant cascade reaction. Finally, it leads to the activation of endogenous antioxidant enzymes such as heme oxygenase-1 (HO-1) and NQO1 [26].

#### 4.2.4. MAPK Signaling Pathway

The sesquiterpenoids in the active components of AOF have inhibitory effects on the phosphorylation of ERK1/2, JNK, and p38 and reduce the expression of pro-inflammatory mediators such as cyclooxygenase-2 (COX-2), inducible iNOS, NO, TNF-α, and IL-6 [23]. Conversely, PCA can activate the ERK1/2, JNK, and p38 pathways, induce the expression of uPA, tissue plasminogen activator (tPA), and MMP2/9, promote the degradation of extracellular matrix (ECM), and enhance the cell migration ability [28]. Although the specific signal transduction of these different active ingredients in the upstream pathways is not yet clear, the advantages of the multi-component and multi-target neuroprotective effects of AOF that they exhibit are beyond dispute.

## 5. Therapeutic Potential of AOF and Its Biologically Active Compounds in Animal Models of Neurological Disorders

As a traditional Chinese medicinal herb, AOF shows potential in clinical applications due to its significant biological activities. With ongoing research into its chemical constituents and mechanisms, AOF holds promise for treating neurological disorders, potentially offering novel therapeutic strategies. Recent advancements in utilizing its bioactive components for neurological diseases are summarized in Table 2.

### 5.1. Therapeutic Applications in AD

AD is a complex neurodegenerative disorder predominantly affecting the elderly, characterized by memory loss, cognitive decline, and behavioral alterations [98]. The pathogenesis of AD involves interactions among diverse biological factors. According to the amyloid cascade hypothesis, Aβ peptides are generated through the proteolytic processing of APP [99]. Under normal conditions, Aβ is efficiently cleared from the brain. In AD, however, an imbalance between Aβ production and clearance leads to its accumulation in the brain, forming amyloid plaques [100]. Aberrant Aβ deposition represents one of the core pathological hallmarks of AD, with soluble Aβ oligomers causing neurotoxicity in early stages [101]. Furthermore, tau protein dysregulation contributes to AD progression. Hyperphosphorylated tau forms neurofibrillary tangles, damaging neuronal structure and function [102].

NKT, a sesquiterpene, is the main compound in AOF [16]. Wang et al. [87] investigated NKT’s effects on cognitive impairment in an LPS-induced AD mouse model. The AD model was established via intraventricular LPS injections and was treated with various NKT doses. Y-maze and Morris water maze tests showed that NKT-treated mice performed significantly better than controls, indicating improved learning and memory. Histopathology revealed alleviated LPS-induced neuronal damage and microglial activation in the hippocampus. WB analysis confirmed that NKT decreased inflammatory mediators IL-1β, IL-6, TNF-α, NLRP3, and NF-κB (p65/Rel A) in the hippocampus.

5-Hydroxymethylfurfural (5-HMF), the primary bioactive compound in the ethanolic extract of AOF, exhibits antioxidant, cytoprotective, anti-myocardial ischemic, and hemorheology-improving effects [103]. Liu et al. [88] investigated its therapeutic potential in an AD mouse model induced by Aβ_1–42_. Mice received intracerebroventricular injections of Aβ_1–42_ to establish the AD model and were treated with 5-HMF at doses of 15 μg/kg and 150 μg/kg for five days. Behavioral tests showed improved performance in locomotor activity, Y-maze, and Morris water maze tasks. 5-HMF improved learning and memory, inhibited β-secretase activity, reduced hippocampal Aβ_1–42_ and MDA levels, and enhanced antioxidant enzyme activity (SOD and GPx). Histopathological analysis confirmed that 5-HMF preserved neuronal integrity in the hippocampal CA1 region, indicating its neuroprotection.

### 5.2. Therapeutic Applications in Depression

Depression, a common mental disorder, threatens global physical and mental health. As one of the most severe diseases of our time, it contributes significantly to rising suicide rates in the 21st century [104]. Key symptoms include persistent low mood, anhedonia, guilt, suicidal thoughts, anxiety, appetite loss, and weight reduction [105]. While the exact mechanisms are unclear, hypotheses involve neuroinflammation, the hypothalamic–pituitary–adrenal (HPA) axis, neurotransmitters, and BDNF [106,107]. Neuroinflammation affects depressive symptoms by altering 5-HT metabolism, HPA axis activity, dopamine metabolism, and oxidative stress [108,109,110]. Therefore, neuroinflammation plays a crucial role in depression. Studies show that overexpression of TLR, particularly TLR4, exacerbates depressive symptoms via inflammatory pathways [111]. TLR4 activation triggers MyD88-dependent signaling, leading to NF-κB nuclear translocation and pro-inflammatory cytokine production [112].

AOVO, rich in sesquiterpenes, exerts significant neuroprotective effects [91]. Wu et al. [91] explored AOVO’s effects on chronic unpredictable mild stress (CUMS)-induced depressive behaviors in mice and its mechanisms. The results showed that AOVO increased mouse activity in the open field test. In the sucrose preference test, compared to controls, the body weight and sucrose preference index improved dose-dependently: 0.50 mL/kg (62.44% body weight increase, 8.98% index improvement), 1.00 mL/kg (127.15%, 29.15%), and 2.00 mL/kg (128.95%, 29.66%). In the novel food suppression test, feeding latency decreased; in the forced swimming test, immobility time reduced, confirming AOVO’s antidepressant effects. Furthermore, AOVO lowered hippocampal pro-inflammatory cytokines (IL-1β, IL-6, TNF-α) and up-regulated serotonin by inhibiting the TLR4-mediated MyD88/NF-κB pathway, reducing inflammation, and protecting hippocampal neurons from CUMS-induced damage.

In the study by Yan et al. [92], CUMS mice were orally administered AOF (10, 20, 40 mg/kg) daily for 21 days. Behavioral tests showed that AOF reduced immobility in the forced swim test and increased sucrose preference. AOF also up-regulated hippocampal BDNF and TrkB, activating downstream signaling (p-CREB and p-Akt), alleviated neuronal damage, and promoted neural plasticity via the BDNF-TrkB pathway. These results indicate that AOF effectively improved depressive behaviors in CUMS mice.

### 5.3. Therapeutic Applications in PD

PD is a neurodegenerative disorder caused by the loss of dopaminergic neurons in the substantia nigra, resulting in motor dysfunction [113] and symptoms such as bradykinesia, tremor, rigidity, postural instability, cognitive impairment, sleep disorders, and mood disturbances [114].

Zhou et al. [115] discovered that oxyphylla A, a component of AOF, possesses therapeutic potential for PD. The deposition of α-synuclein (α-syn) in neurons is a critical pathological feature of PD. In vitro, doxorubicin-induced PC12 cells overexpressing α-syn were used as a PD model. Oxyphylla A treatment significantly promoted α-syn degradation and enhanced PSMB8 expression and proteasome activity via the PKA/Akt/mTOR pathway. In in vivo experiments, 30 mg/kg oxyphylla A administered to A53T α-syn transgenic mice for four weeks prevented dopaminergic neuron loss, protecting against α-syn-induced neurotoxicity. Thus, oxyphylla A serves as a candidate drug for PD.

Furthermore, the therapeutic potential of oxyphylla A for PD was demonstrated in zebrafish models. Chen et al. [116] isolated oxyphylla A from AOF using chiral High Performance Liquid Chromatography–Multiple Reaction Monitoring Mass Spectrometry and administered it to MPTP-induced PD zebrafish. Low-dose oxyphylla A exerted neuroprotective effects, and high-dose treatment did not cause neurotoxicity in zebrafish larvae. These results support further exploration of oxyphylla A as a plant-based food for preventing neurological disorders.

### 5.4. Therapeutic Effects in Cerebral Ischemic Injury

He et al. [117] studied a rat model of surgically induced cerebral ischemic injury, administering AOF extracts and coumaric acid as treatments. Morris water maze tests demonstrated enhanced spatial learning and memory, as evidenced by a shortened escape latency and increased platform crossings. Further mechanistic analyses revealed that both treatments up-regulated BDNF and TrkB expression, activating downstream signaling (p-CREB and p-Akt) to promote hippocampal neurogenesis. These findings suggest that AOF and coumaric acid mitigate post-ischemic cognitive dysfunction through BDNF signaling and neurogenesis enhancement, providing novel evidence for developing natural neuroprotective agents.

## 6. Summary and Expectations

AOF is a commonly used Chinese herb with dual properties as both medicine and food. It is also one of the “Four Major Southern Herbs” in China, alongside *Alpiniae oxyphyllae* Fructus, Amomi Fructus, *Morindae Officinalis* Radix, and Arecae Semen. Historically, it has been widely used in TCM scientific research, which has revealed its neuroprotective potential, as attributed to bioactive compounds like chrysin, PCA, and NKT. These components reduce inflammation, oxidative stress, and apoptosis while promoting cell migration, proliferation, and metabolic regulation. Studies suggest that AOF and its constituents, such as NKT, 5-HMF, and chrysin, may offer therapeutic benefits for neurological disorders, including AD, PD, and depression.

Although AOF shows promise for treating neurological diseases, current research is limited and lacks depth. Most studies focus on its anti-cancer and antiviral properties, with few addressing neurological disorders, especially specific diseases. Existing research isolates individual components to analyze their effects but overlooks synergistic interactions among multiple components on neurological diseases. Traditional Chinese medicine emphasizes holistic approaches, yet experimental studies on AOF’s collaborative effects with other drugs for neurological diseases are scarce. There is an urgent need for such studies to advance clinical applications. Given its multi-target and multi-pathway characteristics, accessibility, and low toxicity, AOF holds potential as a novel neuroprotective agent, providing a valuable therapeutic option for neurodegenerative disorders.

## Figures and Tables

**Table 1:** Research progress on neuroprotective effects of AOF extract and its bioactive components.

Subject	Test Model	Research Results	Effect	Ref.
Sesquiterpenoids in AOF	LPS-stimulated BV-2 microglia	It can significantly inhibit the secretion of nitric oxide (NO), tumor necrosis factor-α (TNF-α), and interleukin-6 (IL-6), down-regulate the expression of inflammation-related genes and proteins, and suppress the phosphorylation of extracellular signal-regulated kinase 1/2 (ERK1/2), c-Jun N-terminal kinase (JNK), and mitogen-activated protein kinase (p38) in the mitogen-activated protein kinase (MAPK) signaling pathway.	anti-inflammatory	[23]
Chrysin, etc.	LPS-stimulated BV-2 microglia	It can promote the transformation of microglia from the pro-inflammatory M1 phenotype to the anti-inflammatory M2 phenotype, reduce the secretion of pro-inflammatory factors (NO, TNF-α, etc.), and increase the expression of anti-inflammatory factors such as interleukin-10 (IL-10), etc.	anti-inflammatory	[24]
AOF extract, PCA, NKT, etc.	Hydrogen peroxide (H_2_O_2_) stimulated PC12 cells	It activates the phosphatidylinositol-3-kinase (PI3K)/serine/threonine-protein kinase B (Akt) signaling pathway, up-regulates the expression of B-cell lymphoma-2 (Bcl-2), inhibits the activation of Bcl-2-associated X protein (BAX) and caspase-3, and simultaneously reduces the reactive oxygen species (ROS) level and enhances the activity of superoxide dismutase (SOD)/catalase (CAT)/glutathione peroxidase (GSH-Px) antioxidant enzymes.	Antioxidant, antiapoptotic	[25]
Oxyphylla A	N2a/APP cells, SAMP8 mice	It can reduce amyloid-β (Aβ) production and improve cognitive deficits in AD models by inhibiting oxidative stress (dependent on the Akt/GSK3β and Nrf2/Keap1/HO-1 pathway).	Antioxidant	[26]
NKT	PC12 cells induced by Aβ_1–42_	The combination of NKT and schisandrin (SCH) can synergistically inhibit Aβ generation and tau phosphorylation through the PI3K/AKT pathway while alleviating oxidative stress and apoptosis.	Antioxidant, antiapoptotic	[27]
PCA	RSC96 Schwann cells	It can significantly promote the migration of Schwann cells by activating the ERK1/2, JNK, and p38 MAPK signaling pathways, promoting the expression and activity of urokinase-type plasminogen activator (uPA), tissue plasminogen activator (tPA), and metalloproteinase 2/9 (MMP2/9).	Promote cell migration	[28]
AOF extract (spray-dried powder)	Sciatic nerve defect model in rats, RSC96 Schwann cells	It can activate the IGF1R-PI3K/Akt signaling pathway and promote the proliferation of Schwann cells, the cell cycle process (transition from G1 phase to S phase), and the expression of cyclins (Cyclin D1, E, and A), thereby promoting sciatic nerve regeneration.	Promote cell proliferation	[29]
AOF extract	APP/PS1 transgenic mice, RSC96 Schwann cells	It can regulate the metabolism of bile acids and sphingolipids in the plasma of mice, improve cognitive function, and reduce the deposition of Aβ in the brain.	Regulate metabolism	[30]
AOF extract	Aβ_1–42_ -induced AD rat model	It can regulate biomarkers related to amino acids, lipids, and energy metabolism (such as arginine, lysophospholipids, and acylcarnitine), improve cognitive function, and reduce Aβ deposition in the brain.	Regulate metabolism	[31]

**Table 2:** Research advances on bioactive components from AOF in the treatment of neurological diseases.

Diseases	Components	Mechanism of Action	Ref.
AD	NKT	It significantly reduced the expression levels of inflammatory factors (IL-1β, IL-6, TNF-α, NOD-like receptor thermal protein domain-associated protein 3 (NLRP3), and NF-κB p65) in the hippocampal region, indicating that its protective effects are mediated through the suppression of neuroinflammation.	[87]
	5-hydroxymethylfurfural	It inhibited β-secretase activity, thereby reducing the levels of Aβ_1–42_ and malondialdehyde (MDA), while enhancing the activity of antioxidant enzymes (SOD, GPx).	[88]
	Chrysin	It reduced the levels of Aβ and phosphorylated tau and exhibited dual inhibitory activity against BACE1 and GSK3β.	[89]
	PCA	It alleviated the inhibitory effect of Aβ_25–35_ on the autophagy–lysosome pathway in primary neurons.	[90]
Depression	*Alpinia oxyphylla* Miq. volatile oil (AOVO)	It inhibited the TLR4-mediated myeloid differentiation primary response gene 88 (MyD88)/NF-κB signaling pathway, significantly reducing the levels of pro-inflammatory cytokines (IL-1β, IL-6, and TNF-α) in the hippocampal region while simultaneously up-regulating serotonin (5-hydroxytryptamine and 5-HT) levels.	[91]
	Flavonoids	It up-regulated the expression of BDNF and its receptor TrkB in the hippocampal region, activating downstream signaling molecules (phosphorylated cAMP response element-binding protein (p-CREB) and p-Akt).	[92]
	Chrysin	It inhibits microglial activation, reducing NLRP3 and IL-1β expression, and blocks Fyn phosphorylation, thereby suppressing the expression of the NLRP3 inflammasome and the NF-κB pathway.	[93]
	NKT	It activated the Keap1/Nrf2/HO-1 antioxidant signaling pathway, enhancing cellular defense mechanisms against oxidative stress.	[94]
PD	Oxyphylla A	It activates the NRF2 pathway, alleviating chemically induced primary neuron damage in vitro, and mitigating chemically induced dopaminergic neuron loss and behavioral deficits in vivo.	[95]
	PCA	It reduces oxidative damage by means of the Polo-like kinase 2/p-GSK3 signaling pathway and alleviates mitochondrial dysfunction via the β/Nrf2 pathway.	[96]
	NKT	It activates the PI3K/Akt signaling pathway to inhibit the expression of MAPK3.	[97]

## Data Availability

No new data were created or analyzed in this study.

## Abbreviations

5-HMF	5-Hydroxymethylfurfural
5-HT	5-hydroxytryptamine
AD	Alzheimer’s disease
AKT	Serine/threonine-protein kinase B
AOF	Alpiniae oxyphyllae Fructus
AOVO	Alpinia oxyphylla Miq. volatile oil
APP	Amyloid precursor protein
Aβ	Amyloid-β
BACE1	β-site amyloid precursor protein cleaving enzyme 1
Bad	Bcl2-associated death promoter
Bak	BCL2 antagonist/killer 1
Bax	Bcl-2-associated X protein
BCL-2	B-cell lymphoma-2
BCL-XL	B-cell lymphoma-extra large
BDNF	Brain-derived neurotrophic factor
CAT	Catalase
CD	Cluster of differentiation
CUMS	Chronic unpredictable mild stress
CNS	Central nervous system
DAG	Diacylglycerol
DAP	DNAX activation protein
ECM	Extracellular matrix
ErK	Extracellular signal-regulated kinase
GPx	Glutathione peroxidase
GSH-Px	Glutathione peroxidase
GSK3β	Glycogen synthase kinase 3β
H_2_O_2_	Hydrogen peroxide
HPA	Hypothalamic–pituitary–adrenal
IGF-1	Insulin-like growth factor 1
IGF-1R	Insulin-like growth factor 1 receptor
IKKα/β	IκB kinase alpha/beta
IL	Interleukin
iNOS	Inducible nitric oxide synthase
IP3	Inositol 1,4,5-trisphosphate
IRAK4	Interleukin-1 receptor-associated kinase 4
IκB	Inhibitor of NF-κB
JNK	c-Jun N-terminal kinase
Keap1	Kelch-like ECH-associated protein 1
LPS	Lipopolysaccharide
MAPK	Mitogen-activated protein kinase
MDA	Malondialdehyde
MDM2	Mouse double minute 2 homolog
MEK	Mitogen-activated protein kinase
MMP	Mitochondrial membrane potential
mPTP	Mitochondrial permeability transition pore
mTOR	Mechanistic target of rapamycin
MyD88	Myeloid differentiation primary response gene 88
NF-κB	Nuclear factor kappa-B
NKT	Nootkatone
NLRP3	NOD-like receptor thermal protein domain-associated protein 3
NO	Nitrogen dioxide
Nrf2	Nuclear factor erythroid 2-related factor 2
P38	p38 mitogen-activated protein kinase
P53	Tumor protein 53
P65/Rel A	RELA proto-oncogene, NF-κB subunit
p-Akt	Phosphorylated Akt
PCA	Protocatechuic acid
PCNA	Proliferating cell nuclear antigen
p-CREB	Phosphorylated cAMP response element-binding protein
PD	Parkinson’s disease
PI3K	Phosphatidylinositol-3-kinase
PIP3	Phosphatidylinositol 3-phosphate
PKC	Protein kinase C
PLCγ	Phospholipase C-gamma
PNS	Peripheral nervous system
Raf	Rapidly accelerated fibrosarcoma
Ras	Rat sarcoma virus oncogene
RNS	Reactive nitrogen species
ROS	Reactive oxygen species
SHC	Src homology 2 domain-containing protein
SOD	Superoxide dismutase
Sos	Son of sevenless
src	Proto-oncogene tyrosine-protein kinase Src
syk	Spleen tyrosine kinase
TAB1/2	TAK1-binding protein 1
TAK1	Transforming growth factor β-activated kinase 1
TCM	Traditional Chinese medicine
TLR	Toll-like receptors
TLR4	Toll-like receptor 4
TNF-α	Tumor necrosis factor-α
tPA	Tissue-type plasminogen activator
TRAF6	TNF receptor-associated factor 6
TREM2	Triggering receptor expressed on myeloid cells 2
TrkB	Tropomyosin receptor kinase B
TSC2	Tuberous sclerosis complex 2
uPA	Urokinase-type plasminogen activator
α-syn	α-synuclein

## References

[1] Wang Y., Wang M., Fan K., Li T., Yan T., Wu B., Bi K., Jia Y. (2018). Protective effects of Alpinae Oxyphyllae Fructus extracts on lipopolysaccharide-induced animal model of Alzheimer’s disease. J. Ethnopharmacol..

[2] Zhang Q., Zheng Y., Hu X., Hu X., Lv W., Lv D., Chen J., Wu M., Song Q., Shentu J. (2018). Ethnopharmacological uses, phytochemistry, biological activities, and therapeutic applications of Alpinia oxyphylla Miquel: A review. J. Ethnopharmacol..

[3] Zuo L., Li J., Xue L., Jia Q., Li Z., Zhang M., Zhao M., Wang M., Kang J., Du S. (2021). Integrated UPLC-MS/MS and UHPLC-Q-orbitrap HRMS Analysis to Reveal Pharmacokinetics and Metabolism of Five Terpenoids from Alpiniae oxyphyllae Fructus in Rats. Curr. Drug Metab..

[4] Li J., Du Q., Li N., Du S., Sun Z. (2021). Alpiniae oxyphyllae Fructus and Alzheimer’s disease: An update and current perspective on this traditional Chinese medicine. Biomed. Pharmacother..

[5] Liao J., Zhao X. (2024). Recent Research Progress on the Chemical Constituents, Pharmacology, and Pharmacokinetics of Alpinae oxyphyllae Fructus. Molecules.

[6] Kirkland A.E., Sarlo G.L., Holton K.F. (2018). The Role of Magnesium in Neurological Disorders. Nutrients.

[7] Nimgampalle M., Chakravarthy H., Sharma S., Shree S., Bhat A.R., Pradeepkiran J.A., Devanathan V. (2023). Neurotransmitter systems in the etiology of major neurological disorders: Emerging insights and therapeutic implications. Ageing Res. Rev..

[8] Park G.W., Kim H., Won S.H., Kim N.H., Choi S.R. (2025). Neurosteroids and neurological disorders. Korean J. Physiol. Pharmacol..

[9] Dash U.C., Bhol N.K., Swain S.K., Samal R.R., Nayak P.K., Raina V., Panda S.K., Kerry R.G., Duttaroy A.K., Jena A.B. (2025). Oxidative stress and inflammation in the pathogenesis of neurological disorders: Mechanisms and implications. Acta Pharm. Sin. B.

[10] Feigin V.L., Vos T., Nichols E., Owolabi M.O., Carroll W.M., Dichgans M., Deuschl G., Parmar P., Brainin M., Murray C. (2020). The global burden of neurological disorders: Translating evidence into policy. Lancet Neurol..

[11] Zhang J., Huang Y., Xu J., Zhao R., Xiong C., Habu J., Wang Y., Luo X. (2022). Global publication trends and research hotspots of curcumin application in tumor: A 20-year bibliometric approach. Front. Oncol..

[12] Jiang S., Liu Y., Zheng H., Zhang L., Zhao H., Sang X., Xu Y., Lu X. (2023). Evolutionary patterns and research frontiers in neoadjuvant immunotherapy: A bibliometric analysis. Int. J. Surg..

[13] Arruda H., Silva E.R., Lessa M., Proença D., Bartholo R. (2022). VOSviewer and Bibliometrix. J. Med. Libr. Assoc..

[14] Zhang J., Liu H., Che T., Zheng Y., Nan X., Wu Z. (2023). Nanomaterials for diabetic wound healing: Visualization and bibliometric analysis from 2011 to 2021. Front. Endocrinol..

[15] Zhang Y., Dong L., Zhang Q., Han J., Sui W., Wei Z., Zhang Z., Tong Y., Wang S., Han F. (2022). Qualitative and quantitative analysis of Alpiniae oxyphyllae Fructus by high-performance liquid chromatography coupled to Fourier transform-ion cyclotron resonance mass spectrometry. J. Sep. Sci..

[16] Xiao T., Pan M., Wang Y., Huang Y., Tsunoda M., Zhang Y., Wang R., Hu W., Yang H., Li L.S. (2023). In vitro bloodbrain barrier permeability study of four main active ingredients from Alpiniae oxyphyllae fructus. J. Pharm. Biomed. Anal..

[17] Zhang Y., Huang Z., Zhou Z., Ma N., Wang R., Chen M., He X., Dong L., Xia Z., Liu Q. (2022). Nootkatone, a Sesquiterpene Ketone From Alpiniae oxyphyllae Fructus, Ameliorates Metabolic-Associated Fatty Liver by Regulating AMPK and MAPK Signaling. Front. Pharmacol..

[18] Yang X., Yang Y., Chen H., Xu T., Li C., Zhou R., Gao L., Han M., He X., Chen Y. (2020). Extraction, isolation, immunoregulatory activity, and characterization of Alpiniae oxyphyllae fructus polysaccharides. Int. J. Biol. Macromol..

[19] Koo B.S., Lee W.C., Chang Y.C., Kim C.H. (2004). Protective effects of alpinae oxyphyllae fructus (Alpinia oxyphylla MIQ) water-extracts on neurons from ischemic damage and neuronal cell toxicity. Phytother. Res..

[20] Qiu C., Mu L., Wang J., Tang R., Hou B., Hu W., Zhang R., Chen X. (2023). Sesquiterpenoids from the fruits of Alpinia oxyphylla Miq. and their neuroprotective effect. Phytochemistry.

[21] Yu X., An L., Wang Y., Zhao H., Gao C. (2003). Neuroprotective effect of Alpinia oxyphylla Miq. fruits against glutamate-induced apoptosis in cortical neurons. Toxicol. Lett..

[22] Xu J., Wang F., Guo J., Xu C., Cao Y., Fang Z., Wang Q. (2020). Pharmacological Mechanisms Underlying the Neuroprotective Effects of Alpinia oxyphylla Miq. on Alzheimer’s Disease. Int. J. Mol. Sci..

[23] Dong J., Zhou M., Pan D.B., Qin Q.Y., Li T., Yao X.S., Li H.B., Yu Y. (2023). Eremophilane and cadinane sesquiterpenoids from the fruits of Alpinia oxyphylla and their anti-inflammatory activities. Food Funct..

[24] Xu M., Yang Y., Peng J., Zhang Y., Wu B., He B., Jia Y., Yan T. (2023). Effects of Alpinae Oxyphyllae Fructus on microglial polarization in a LPS-induced BV2 cells model of neuroinflammation via TREM2. J. Ethnopharmacol..

[25] Li R., Wang L., Zhang Q., Duan H., Qian D., Yang F., Xia J. (2022). Alpiniae oxyphyllae fructus possesses neuroprotective effects on H_2_O_2_ stimulated PC12 cells via regulation of the PI3K/Akt signaling Pathway. Front. Pharmacol..

[26] Bian Y., Chen Y., Wang X., Cui G., Ung C.O.L., Lu J.-H., Cong W., Tang B., Lee S.M.-Y. (2021). Oxyphylla A ameliorates cognitive deficits and alleviates neuropathology via the Akt-GSK3β and Nrf2-Keap1-HO-1 pathways in vitro and in vivo murine models of Alzheimer’s disease. J. Adv. Res..

[27] Qi Y., Cheng X., Gong G., Yan T., Du Y., Wu B., Bi K., Jia Y. (2020). Synergistic neuroprotective effect of schisandrin and nootkatone on regulating inflammation, apoptosis and autophagy via the PI3K/AKT pathway. Food Funct..

[28] Ju D.-T., Kuo W.-W., Ho T.-J., Paul C.R., Kuo C.-H., Viswanadha V.P., Lin C.-C., Chen Y.-S., Chang Y.-M., Huang C.-Y. (2015). Protocatechuic Acid from Alpinia oxyphylla Induces Schwann Cell Migration via ERK1/2, JNK and p38 Activation. Am. J. Chin. Med..

[29] Chang Y.-M., Chang H.-H., Tsai C.-C., Lin H.-J., Ho T.-J., Ye C.-X., Chiu P.-L., Chen Y.-S., Chen R.-J., Huang C.-Y. (2017). Alpinia oxyphylla Miq. fruit extract activates IGFR-PI3K/Akt signaling to induce Schwann cell proliferation and sciatic nerve regeneration. BMC Complement. Altern. Med..

[30] Zhou S., Liu L., Zhang Y., Zhang Z., Li H., Fan F., He J., Kang J., Zuo L. (2023). Integrated untargeted and targeted metabolomics to reveal therapeutic effect and mechanism of Alpiniae oxyphyllae fructus on Alzheimer’s disease in APP/PS1 mice. Front. Pharmacol..

[31] Duncan R.E., Sun Z., Zhang Y., Zhang M., Zhou S., Cheng W., Xue L., Zhou P., Li X., Zhang Z. (2023). Integrated brain and plasma dual-channel metabolomics to explore the treatment effects of Alpinia oxyphyllaFructus on Alzheimer’s disease. PLoS ONE.

[32] Singh N., Baby D., Rajguru J.P., Patil P.B., Thakkannavar S.S., Pujari V.B. (2019). Inflammation and cancer. Ann. Afr. Med..

[33] Chen Y., Zhang Y., Wang J., Li S., Wang Y., Zhang Z., Zhang J., Xin C., Wang Y., Rong P. (2023). Anti-neuroinflammation effects of transcutaneous auricular vagus nerve stimulation against depression-like behaviors via hypothalamic α7nAchR/JAK2/STAT3/NF-κB pathway in rats exposed to chronic unpredictable mild stress. CNS Neurosci. Ther..

[34] Hong X., Chen T., Liu Y., Li J., Huang D., Ye K., Liao W., Wang Y., Liu M., Luan P. (2025). Design, current states, and challenges of nanomaterials in anti-neuroinflammation: A perspective on Alzheimer’s disease. Ageing Res. Rev..

[35] Kwon H.S., Koh S.-H. (2020). Neuroinflammation in neurodegenerative disorders: The roles of microglia and astrocytes. Transl. Neurodegener..

[36] Beltran-Velasco A.I., Clemente-Suárez V.J. (2025). Impact of Peripheral Inflammation on Blood-Brain Barrier Dysfunction and Its Role in Neurodegenerative Diseases. Int. J. Mol. Sci..

[37] Zhang X.W., Feng N., Liu Y.C., Guo Q., Wang J.K., Bai Y.Z., Ye X.M., Yang Z., Yang H., Liu Y. (2022). Neuroinflammation inhibition by small-molecule targeting USP7 noncatalytic domain for neurodegenerative disease therapy. Sci. Adv..

[38] Deczkowska A., Keren-Shaul H., Weiner A., Colonna M., Schwartz M., Amit I. (2018). Disease-Associated Microglia: A Universal Immune Sensor of Neurodegeneration. Cell.

[39] Yoshikawa T., You F. (2024). Oxidative Stress and Bio-Regulation. Int. J. Mol. Sci..

[40] Chang W.L., Ko C.H. (2023). The Role of Oxidative Stress in Vitiligo: An Update on Its Pathogenesis and Therapeutic Implications. Cells.

[41] Veluthakal R., Esparza D., Hoolachan J.M., Balakrishnan R., Ahn M., Oh E., Jayasena C.S., Thurmond D.C. (2024). Mitochondrial Dysfunction, Oxidative Stress, and Inter-Organ Miscommunications in T2D Progression. Int. J. Mol. Sci..

[42] Zhang Q., Shen X., Yuan X., Huang J., Zhu Y., Zhu T., Zhang T., Wu H., Wu Q., Fan Y. (2024). Lipopolysaccharide binding protein resists hepatic oxidative stress by regulating lipid droplet homeostasis. Nat. Commun..

[43] Han Y., Zhang M., Yu S., Jia L. (2025). Oxidative Stress in Pediatric Asthma: Sources, Mechanisms, and Therapeutic Potential of Antioxidants. Front. Biosci. Landmark Ed..

[44] Choi E.H., Kim M.H., Park S.J. (2024). Targeting Mitochondrial Dysfunction and Reactive Oxygen Species for Neurodegenerative Disease Treatment. Int. J. Mol. Sci..

[45] Gogna T., Housden B.E., Houldsworth A. (2024). Exploring the Role of Reactive Oxygen Species in the Pathogenesis and Pathophysiology of Alzheimer’s and Parkinson’s Disease and the Efficacy of Antioxidant Treatment. Antioxidants.

[46] Li Y., Zhang W., Zhang Q., Li Y., Xin C., Tu R., Yan H. (2025). Oxidative stress of mitophagy in neurodegenerative diseases: Mechanism and potential therapeutic targets. Arch. Biochem. Biophys..

[47] Xiao C.L., Lai H.T., Zhou J.J., Liu W.Y., Zhao M., Zhao K. (2025). Nrf2 Signaling Pathway: Focus on Oxidative Stress in Spinal Cord Injury. Mol. Neurobiol..

[48] Wen P., Sun Z., Gou F., Wang J., Fan Q., Zhao D., Yang L. (2025). Oxidative stress and mitochondrial impairment: Key drivers in neurodegenerative disorders. Ageing Res. Rev..

[49] Üremiş N., Üremiş M.M. (2025). Oxidative/Nitrosative Stress, Apoptosis, and Redox Signaling: Key Players in Neurodegenerative Diseases. J. Biochem. Mol. Toxicol..

[50] Tamagno E., Guglielmotto M., Vasciaveo V., Tabaton M. (2021). Oxidative Stress and Beta Amyloid in Alzheimer’s Disease. Which Comes First: The Chicken or the Egg?. Antioxidants.

[51] Wang J., Liu M., Zhao J., Hu P., Gao L., Tian S., Zhang J., Liu H., Xu X., He Z. (2025). Oxidative stress and dysregulated long noncoding RNAs in the pathogenesis of Parkinson’s disease. Biol. Res..

[52] Liu Y., Shen X., Zhang Y., Zheng X., Cepeda C., Wang Y., Duan S., Tong X. (2023). Interactions of glial cells with neuronal synapses, from astrocytes to microglia and oligodendrocyte lineage cells. Glia.

[53] Bunge R.P. (1993). Expanding roles for the Schwann cell: Ensheathment, myelination, trophism and regeneration. Curr. Opin. Neurobiol..

[54] Kampanis V., Tolou-Dabbaghian B., Zhou L., Roth W., Puttagunta R. (2020). Cyclic Stretch of Either PNS or CNS Located Nerves Can Stimulate Neurite Outgrowth. Cells.

[55] Cárdenas A., Kong M., Alvarez A., Maldonado H., Leyton L. (2014). Signaling pathways involved in neuron-astrocyte adhesion and migration. Curr. Mol. Med..

[56] Su Y., Wang X., Yang Y., Chen L., Xia W., Hoi K.K., Li H., Wang Q., Yu G., Chen X. (2023). Astrocyte endfoot formation controls the termination of oligodendrocyte precursor cell perivascular migration during development. Neuron.

[57] Oshima K., Yoshinaga S., Kitazawa A., Hirota Y., Nakajima K., Kubo K.I. (2023). A Unique “Reversed” Migration of Neurons in the Developing Claustrum. J. Neurosci..

[58] Matsumoto M., Matsushita K., Hane M., Wen C., Kurematsu C., Ota H., Bang Nguyen H., Quynh Thai T., Herranz-Pérez V., Sawada M. (2024). Neuraminidase inhibition promotes the collective migration of neurons and recovery of brain function. EMBO Mol. Med..

[59] Wang Y.T., Yuan H. (2022). Research progress of endogenous neural stem cells in spinal cord injury. Ibrain.

[60] Rueger M.A., Androutsellis-Theotokis A. (2013). Identifying endogenous neural stem cells in the adult brain in vitro and in vivo: Novel approaches. Curr. Pharm. Des..

[61] Mubuchi A., Takechi M., Nishio S., Matsuda T., Itoh Y., Sato C., Kitajima K., Kitagawa H., Miyata S. (2024). Assembly of neuron- and radial glial-cell-derived extracellular matrix molecules promotes radial migration of developing cortical neurons. Elife.

[62] Taylor K.R., Monje M. (2023). Neuron-oligodendroglial interactions in health and malignant disease. Nat. Rev. Neurosci..

[63] Ott C.M., Constable S., Nguyen T.M., White K., Lee W.A., Lippincott-Schwartz J., Mukhopadhyay S. (2024). Permanent deconstruction of intracellular primary cilia in differentiating granule cell neurons. J. Cell Biol..

[64] Zhang X., Duan S., Apostolou P.E., Wu X., Watanabe J., Gallitto M., Barron T., Taylor K.R., Woo P.J., Hua X. (2024). CHD2 Regulates Neuron-Glioma Interactions in Pediatric Glioma. Cancer Discov..

[65] Araragi N., Petermann M., Suzuki M., Larkum M., Mosienko V., Bader M., Alenina N., Klempin F. (2025). Acute Optogenetic Stimulation of Serotonin Neurons Reduces Cell Proliferation in the Dentate Gyrus of Mice. ACS Chem. Neurosci..

[66] Cai R., Wang Y., Huang Z., Zou Q., Pu Y., Yu C., Cai Z. (2021). Role of RhoA/ROCK signaling in Alzheimer’s disease. Behav. Brain Res..

[67] Medd M.M., Yon J.E., Dong H. (2025). RhoA/ROCK/GSK3β Signaling: A Keystone in Understanding Alzheimer’s Disease. Curr. Issues Mol. Biol..

[68] Ramakrishan P., Rajangam J., Mahinoor S.S., Bisht S., Mekala S., Upadhyay D.K., Solomon V.R., Sabarees G., Pelluri R. (2025). Unveiling the mTOR pathway modulation by SGLT2 inhibitors: A novel approach to Alzheimer’s disease in type 2 diabetes. Metab. Brain Dis..

[69] Salzer J., Feltri M.L., Jacob C. (2024). Schwann Cell Development and Myelination. Cold Spring Harb. Perspect. Biol..

[70] Anton E.S., Sandrock A.W., Matthew W.D. (1994). Merosin promotes neurite growth and Schwann cell migration in vitro and nerve regeneration in vivo: Evidence using an antibody to merosin, ARM-1. Dev. Biol..

[71] Torigoe K., Tanaka H.F., Takahashi A., Awaya A., Hashimoto K. (1996). Basic behavior of migratory Schwann cells in peripheral nerve regeneration. Exp. Neurol..

[72] O’Brien A.L., West J.M., Saffari T.M., Nguyen M., Moore A.M. (2022). Promoting Nerve Regeneration: Electrical Stimulation, Gene Therapy, and Beyond. Physiology.

[73] Nocera G., Jacob C. (2020). Mechanisms of Schwann cell plasticity involved in peripheral nerve repair after injury. Cell Mol. Life Sci..

[74] Tagliatti E., Desiato G., Mancinelli S., Bizzotto M., Gagliani M.C., Faggiani E., Hernández-Soto R., Cugurra A., Poliseno P., Miotto M. (2024). Trem2 expression in microglia is required to maintain normal neuronal bioenergetics during development. Immunity.

[75] Wang Z., Zhang S., Cheng R., Jiang A., Qin X. (2024). Knockdown of RGMA improves ischemic stroke via Reprogramming of Neuronal Metabolism. Free Radic. Biol. Med..

[76] Qiu S., Wu Q., Wang H., Liu D., Chen C., Zhu Z., Zheng H., Yang G., Li L., Yang M. (2024). AZGP1 in POMC neurons modulates energy homeostasis and metabolism through leptin-mediated STAT3 phosphorylation. Nat. Commun..

[77] Nixon R.A. (2024). Autophagy-lysosomal-associated neuronal death in neurodegenerative disease. Acta Neuropathol..

[78] He K., Zhao Z., Zhang J., Li D., Wang S., Liu Q. (2024). Cholesterol Metabolism in Neurodegenerative Diseases. Antioxid. Redox Signal..

[79] Ma X., Di Q., Li X., Zhao X., Zhang R., Xiao Y., Li X., Wu H., Tang H., Quan J. (2022). Munronoid I Ameliorates DSS-Induced Mouse Colitis by Inhibiting NLRP3 Inflammasome Activation and Pyroptosis Via Modulation of NLRP3. Front. Immunol..

[80] Jiang S., Li H., Zhang L., Mu W., Zhang Y., Chen T., Wu J., Tang H., Zheng S., Liu Y. (2024). Generic Diagramming Platform (GDP): A comprehensive database of high-quality biomedical graphics. Nucleic Acids Research.

[81] Peng X., Guo H., Zhang X., Yang Z., Ruganzu J.B., Yang Z., Wu X., Bi W., Ji S., Yang W. (2023). TREM2 Inhibits Tau Hyperphosphorylation and Neuronal Apoptosis via the PI3K/Akt/GSK-3β Signaling Pathway In vivo and In vitro. Mol. Neurobiol..

[82] Qin Q., Teng Z., Liu C., Li Q., Yin Y., Tang Y. (2021). TREM2, microglia, and Alzheimer’s disease. Mech. Ageing Dev..

[83] Nugent A.A., Lin K., van Lengerich B., Lianoglou S., Przybyla L., Davis S.S., Llapashtica C., Wang J., Kim D.J., Xia D. (2020). TREM2 Regulates Microglial Cholesterol Metabolism upon Chronic Phagocytic Challenge. Neuron.

[84] Rachmian N., Medina S., Cherqui U., Akiva H., Deitch D., Edilbi D., Croese T., Salame T.M., Ramos J.M.P., Cahalon L. (2024). Identification of senescent, TREM2-expressing microglia in aging and Alzheimer’s disease model mouse brain. Nat. Neurosci..

[85] Wang S., Sudan R., Peng V., Zhou Y., Du S., Yuede C.M., Lei T., Hou J., Cai Z., Cella M. (2022). TREM2 drives microglia response to amyloid-β via SYK-dependent and -independent pathways. Cell.

[86] Miao J., Zhang Y., Su C., Zheng Q., Guo J. (2025). Insulin-Like Growth Factor Signaling in Alzheimer’s Disease: Pathophysiology and Therapeutic Strategies. Mol. Neurobiol..

[87] Wang Y., Wang M., Xu M., Li T., Fan K., Yan T., Xiao F., Bi K., Jia Y. (2018). Nootkatone, a neuroprotective agent from Alpiniae oxyphyllae Fructus, improves cognitive impairment in lipopolysaccharide-induced mouse model of Alzheimer’s disease. Int. Immunopharmacol..

[88] Liu A., Zhao X., Li H., Liu Z., Liu B., Mao X., Guo L., Bi K., Jia Y. (2014). 5-Hydroxymethylfurfural, an antioxidant agent from Alpinia oxyphylla Miq. improves cognitive impairment in Aβ 1-42 mouse model of Alzheimer’s disease. Int. Immunopharmacol..

[89] Zhang Z., Li R., Zhou Y., Huang S., Hou Y., Pei G. (2025). Dietary Flavonoid Chrysin Functions as a Dual Modulator to Attenuate Amyloid-β and Tau Pathology in the Models of Alzheimer’s Disease. Mol. Neurobiol..

[90] Chi F., Zhang G., Ren N., Zhang J., Du F., Zheng X., Zhang C., Lin Z., Li R., Shi X. (2022). The anti-alcoholism drug disulfiram effectively ameliorates ulcerative colitis through suppressing oxidative stresses-associated pyroptotic cell death and cellular inflammation in colonic cells. Int. Immunopharmacol..

[91] Wu B., Gan A., Wang R., Lin F., Yan T., Jia Y. (2023). Alpinia oxyphylla Miq. volatile oil ameliorates depressive behaviors and inhibits neuroinflammation in CUMS-exposed mice by inhibiting the TLR4-medicated MyD88/NF-κB signaling pathway. J. Chem. Neuroanat..

[92] Yan T., Wu B., Liao Z.Z., Liu B., Zhao X., Bi K.S., Jia Y. (2016). Brain-derived Neurotrophic Factor Signaling Mediates the Antidepressant-like Effect of the Total Flavonoids of Alpiniae oxyphyllae Fructus in Chronic Unpredictable Mild Stress Mice. Phytother. Res..

[93] Cui L.J., Yuan W., Chen F.Y., Wang Y.X., Li Q.M., Lin C., Miao X.P. (2022). Pectic polysaccharides ameliorate the pathology of ulcerative colitis in mice by reducing pyroptosis. Ann. Transl. Med..

[94] Yan T., Li F., Xiong W., Wu B., Xiao F., He B., Jia Y. (2021). Nootkatone improves anxiety- and depression-like behavior by targeting hyperammonemia-induced oxidative stress in D-galactosamine model of liver injury. Environ. Toxicol..

[95] Li G., Zhang Z., Quan Q., Jiang R., Szeto S.S., Yuan S., Wong W.T., Lam H.H., Lee S.M., Chu I.K. (2016). Discovery, Synthesis, and Functional Characterization of a Novel Neuroprotective Natural Product from the Fruit of Alpinia oxyphylla for use in Parkinson’s Disease Through LC/MS-Based Multivariate Data Analysis-Guided Fractionation. J. Proteome Res..

[96] Guo C., Zhu J., Wang J., Duan J., Ma S., Yin Y., Quan W., Zhang W., Guan Y., Ding Y. (2019). Neuroprotective effects of protocatechuic aldehyde through PLK2/p-GSK3β/Nrf2 signaling pathway in both in vivo and in vitro models of Parkinson’s disease. Aging.

[97] Yao Z., Li J., Bian L., Li Q., Wang X., Yang X., Wei X., Wan G., Wang Y., Shi J. (2022). Nootkatone alleviates rotenone-induced Parkinson’s disease symptoms through activation of the PI3K/Akt signaling pathway. Phytother. Res..

[98] Graff-Radford J., Yong K.X.X., Apostolova L.G., Bouwman F.H., Carrillo M., Dickerson B.C., Rabinovici G.D., Schott J.M., Jones D.T., Murray M.E. (2021). New insights into atypical Alzheimer’s disease in the era of biomarkers. Lancet Neurol..

[99] Twarowski B., Herbet M. (2023). Inflammatory Processes in Alzheimer’s Disease-Pathomechanism, Diagnosis and Treatment: A Review. Int. J. Mol. Sci..

[100] Liu E., Zhang Y., Wang J.Z. (2024). Updates in Alzheimer’s disease: From basic research to diagnosis and therapies. Transl. Neurodegener..

[101] Rostagno A.A. (2022). Pathogenesis of Alzheimer’s Disease. Int. J. Mol. Sci..

[102] Athar T., Al Balushi K., Khan S.A. (2021). Recent advances on drug development and emerging therapeutic agents for Alzheimer’s disease. Mol. Biol. Rep..

[103] Chen C., Lv M., Hu H., Huai L., Zhu B., Fan S., Wang Q., Zhang J. (2024). 5-Hydroxymethylfurfural and its Downstream Chemicals: A Review of Catalytic Routes. Adv. Mater..

[104] Hao Y., Ge H., Sun M., Gao Y. (2019). Selecting an Appropriate Animal Model of Depression. Int. J. Mol. Sci..

[105] Zhang Y., Long Y., Yu S., Li D., Yang M., Guan Y., Zhang D., Wan J., Liu S., Shi A. (2021). Natural volatile oils derived from herbal medicines: A promising therapy way for treating depressive disorder. Pharmacol. Res..

[106] Martins J., Brijesh S. (2018). Phytochemistry and pharmacology of anti-depressant medicinal plants: A review. Biomed. Pharmacother..

[107] Yan T., Nian T., Liao Z., Xiao F., Wu B., Bi K., He B., Jia Y. (2020). Antidepressant effects of a polysaccharide from okra (Abelmoschus esculentus (L) Moench) by anti-inflammation and rebalancing the gut microbiota. Int. J. Biol. Macromol..

[108] Chen B., Li J., Xie Y., Ming X., Li G., Wang J., Li M., Li X., Xiong L. (2019). Cang-ai volatile oil improves depressive-like behaviors and regulates DA and 5-HT metabolism in the brains of CUMS-induced rats. J. Ethnopharmacol..

[109] Yan T., Sun Y., Xiao F., Wu B., Bi K., He B., Jia Y. (2019). Schisandrae Chinensis Fructus inhibits behavioral deficits induced by sleep deprivation and chronic unpredictable mild stress via increased signaling of brain-derived neurotrophic factor. Phytother. Res..

[110] Yan T., He B., Wan S., Xu M., Yang H., Xiao F., Bi K., Jia Y. (2017). Antidepressant-like effects and cognitive enhancement of Schisandra chinensis in chronic unpredictable mild stress mice and its related mechanism. Sci. Rep..

[111] Figueroa-Hall L.K., Paulus M.P., Savitz J. (2020). Toll-Like Receptor Signaling in Depression. Psychoneuroendocrinology.

[112] Zhao J., Bi W., Zhang J., Xiao S., Zhou R., Tsang C.K., Lu D., Zhu L. (2020). USP8 protects against lipopolysaccharide-induced cognitive and motor deficits by modulating microglia phenotypes through TLR4/MyD88/NF-κB signaling pathway in mice. Brain Behav. Immun..

[113] Hayes M.T. (2019). Parkinson’s Disease and Parkinsonism. Am. J. Med..

[114] Bloem B.R., Okun M.S., Klein C. (2021). Parkinson’s disease. Lancet.

[115] Zhou H., Li S., Li C., Yang X., Li H., Zhong H., Lu J.H., Lee S.M. (2020). Oxyphylla A Promotes Degradation of α-Synuclein for Neuroprotection via Activation of Immunoproteasome. Aging Dis..

[116] Chen Y., Li G., Law H.C.H., Chen H., Lee S.M. (2020). Determination of Oxyphylla A Enantiomers in the Fruits of Alpinia oxyphylla by a Chiral High-Performance Liquid Chromatography-Multiple Reaction Monitoring-Mass Spectrometry Method and Comparison of Their In Vivo Biological Activities. J. Agric. Food Chem..

[117] He Y., Chen S., Tsoi B., Qi S., Gu B., Wang Z., Peng C., Shen J. (2020). Alpinia oxyphylla Miq. and Its Active Compound P-Coumaric Acid Promote Brain-Derived Neurotrophic Factor Signaling for Inducing Hippocampal Neurogenesis and Improving Post-cerebral Ischemic Spatial Cognitive Functions. Front. Cell Dev. Biol..

